# DNA Functional Nanomaterials for Controlled Delivery of Nucleic Acid-Based Drugs

**DOI:** 10.3389/fbioe.2021.720291

**Published:** 2021-08-17

**Authors:** Zhaoyue Lv, Yi Zhu, Feng Li

**Affiliations:** Key Laboratory of Systems Bioengineering (MOE), Frontiers Science Center for Synthetic Biology, School of Chemical Engineering and Technology, Tianjin University, Tianjin, China

**Keywords:** nucleic acid-based drugs, drug delivery, DNA assembly, DNA nanomaterials, DNA nanotechnology

## Abstract

Nucleic acid-based drugs exhibited great potential in cancer therapeutics. However, the biological instability of nucleic acid-based drugs seriously hampered their clinical applications. Efficient *in vivo* delivery is the key to the clinical application of nucleic acid-based drugs. As a natural biological macromolecule, DNA has unique properties, such as excellent biocompatibility, molecular programmability, and precise assembly controllability. With the development of DNA nanotechnology, DNA nanomaterials have demonstrated significant advantages as delivery vectors of nucleic acid-based drugs by virtue of the inherent nucleic acid properties. In this study, the recent progress in the design of DNA-based nanomaterials for nucleic acid delivery is summarized. The DNA nanomaterials are categorized according to the components including pure DNA nanomaterials, DNA-inorganic hybrid nanomaterials, and DNA-organic hybrid nanomaterials. Representative applications of DNA nanomaterials in the controlled delivery of nucleic acid-based drugs are exemplified to show how DNA nanomaterials are rationally and exquisitely designed to address application issues in cancer therapy. At the end of this study, the challenges and future development of DNA nanomaterials are discussed.

## Introduction

With the development of genomics and the elucidation of the genetic mechanism of cancer pathogenesis, gene therapy has been a promising therapeutic strategy for a variety of diseases, such as genetic disorders and cancers (Vaughan et al., 2020). Nucleic acid-based drugs mainly include plasmid DNA, antisense oligonucleotides (ASOs), small interfering RNA (siRNA), single-guided RNA (sgRNA), messenger RNA (mRNA), and immunostimulatory nucleic acids (Jiang and Thayumanavan, 2020; Vaughan et al., 2020). Intracellular nucleic acid delivery allows to reprogram cells at the gene level and provides an effective strategy to control protein generation, thus realizing precise regulation on cell functions (Vaughan et al., 2020). Compared with small molecule therapeutics, nucleic acid drugs were hardly taken up by cells due to their large molecular weight and hydrophilic nature. Moreover, nucleic acid drugs could be rapidly degraded by nucleases in the plasma with half-lives of several minutes, causing off-target and immune reactions (Jiang and Thayumanavan, 2020). Therefore, nanocarriers are needed to improve the stability of nucleic acid drugs in the plasma and further facilitate their cellular uptake. In recent years, significant progress has been made in developing nanocarriers for nucleic acid delivery. In particular, DNA-based nanomaterials are emerging as nucleic acid delivery vectors and have been extensively explored.

DNA, which is traditionally regarded as a genetic molecule, possesses excellent sequence programmability, precise molecular recognition, and abundant biological functions (Li F. et al., 2019; Dong et al., 2020). In 1953, Waston and Crick first reported the double-helix structure of DNA, leading to an extensive study on the structure of DNA (Li F. et al., 2019; Zhang et al., 2019). Conventional Watson-Crick base pairing refers to cytosine[C] and guanine[G] pairing, and adenine [A] and thymine [T] pairing (Zhang et al., 2019). In the early 1980s, Seeman initially proposed that DNA could function as building tiles for precisely constructing ordered nanostructure based on the Watson-Crick base pairing principle, opening a new frontier of DNA nanotechnology (Yuan et al., 2019; Dong et al., 2020). Owing to its intrinsic excellent biocompatibility, biodegradability, addressability, and low toxicity, DNA-based nanomaterials showed a unique advantage in disease diagnosis and cancer therapy (Wu et al., 2020). In particular, the excellent sequence programmability of DNA endowed the DNA nanomaterials with dynamic assembling behaviors, which facilitated spatiotemporally controllable assembly and release of nucleic acid drugs.

**FIGURE 1:**
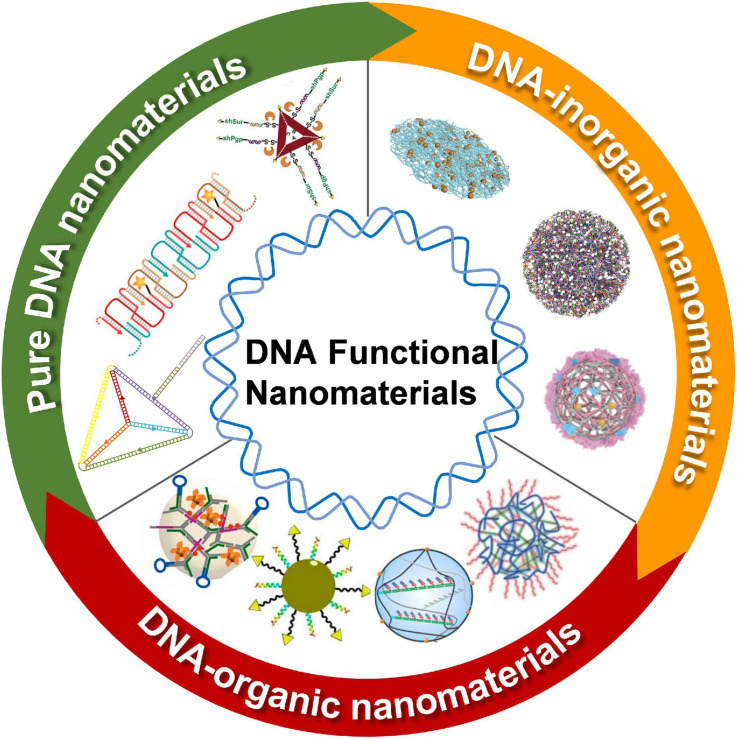
Schematic illustration of DNA functional nanomaterials for nucleic acid-based drugs delivery. DNA functional nanomaterials were developed into three major categories: pure DNA nanomaterials (marked with green), DNA-inorganic nanomaterials (marked with orange), and DNA-inorganic nanomaterials (marked with red). Images in pure DNA nanomaterials section (clockwise); Adapted with permission from Lee et al. (2012). Adapted with permission from Chen et al. (2015). Copyright 2015, American Chemical Society. Adapted with permission from Liu et al. (2018). Copyright 2018, Wiley-VCH. Images in DNA-inorganic nanomaterials section (clockwise); pure DNA nanomaterials (clockwise); Adapted with permission from Huo et al. (2019). Adapted with permission from Li M. et al. (2019). Copyright 2019, Wiley-VCH. Adapted with permission from Wang et al. (2021). Copyright 2021, Wiley-VCH. Images in DNA-organic nanomaterials section (clockwise); Adapted with permission from Ding et al. (2018). Copyright 2018, Wiley-VCH. Adapted with permission from Li et al. (2021). Copyright 2021, Nature Publishing Group. Adapted with permission fromLiu et al., 2019. Copyright 2019, American Chemical Society. Adapted with permission from Han et al. (2021a). Copyright 2021, Elsevier Publishing Group.

In this study, the recent progress on DNA-based nanomaterials for nucleic acid delivery is summarized. According to the components, the DNA nanomaterials are categorized including pure DNA nanomaterials, DNA-inorganic hybrid nanomaterials, and DNA-organic hybrid nanomaterials (Figure 1). Representative applications of the DNA nanomaterials in the controlled delivery of nucleic acid-based drugs are exemplified to show how DNA nanomaterials are rationally and exquisitely designed to address nucleic acid drug delivery issues for cancer therapy. At the end of this study, the challenges and prospects of DNA nanomaterials for the delivery of nucleic acid-based drugs are fully discussed.

## Prue DNA Nanomaterials

**FIGURE 2:**
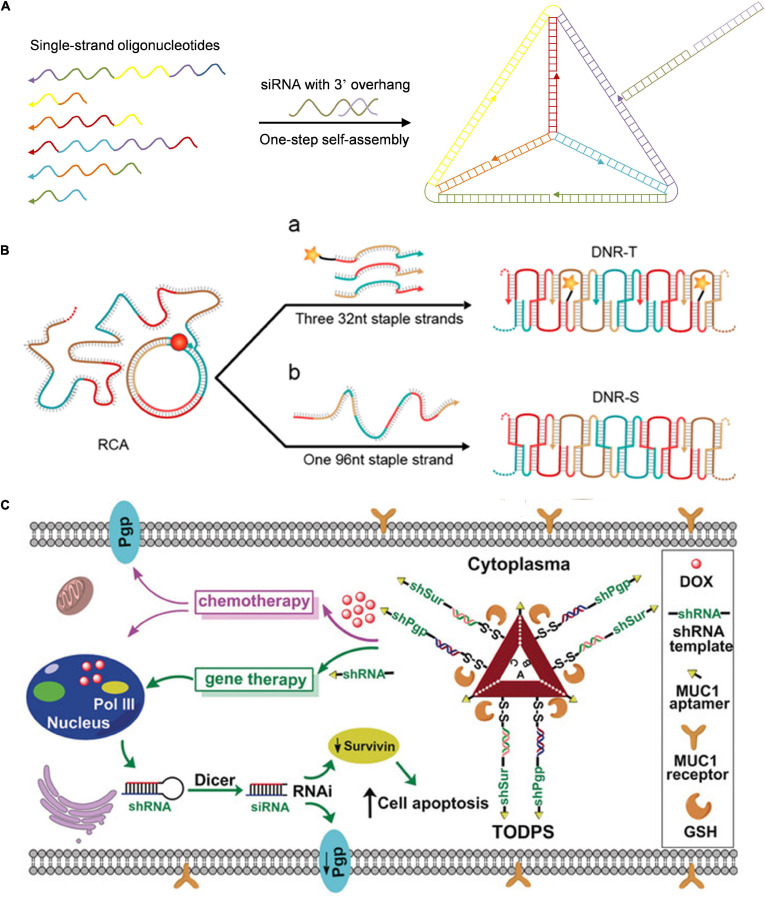
Pure DNA nanoassembly for nucleic acid-based drugs delivery. **(A)** DNA tetrahedron for targeted *in vivo* siRNA delivery. Adapted with permission from ref. Lee et al. (2012). **(B)** DNA nanoribbons for siRNA delivery. Adapted with permission from ref. Chen et al. (2015). Copyright 2015, American Chemical Society. **(C)** DNA origami for synergistic RNAi-/chemotherapy. Adapted with permission from ref. Liu et al. (2018). Copyright 2018, Wiley-VCH.

Pure DNA nanomaterials are primarily constructed *via* self-assembly and DNA amplification technology (Zhang et al., 2019). Pure DNA nanomaterials take full advantage of self-assembly capability between DNA building blocks and nucleic acid drugs to realize controlled assembly and release of nucleic acid drugs as designed. For example, Lee et al. (2012) prepared DNA tetrahedral nanoparticles (NPs) *via* programmable self-assembly of short DNA fragments and therapeutic siRNAs (Figure 2A). Li et al. (2015) constructed a size-controllable and stimuli-responsive DNA nanohydrogel *via* self-assembly of three kinds of DNA building units for enhanced antisense DNA delivery. Long single-stranded DNA (ssDNA) produced by the rolling circle amplification (RCA) reaction was also used to construct DNA nanoribbons for intracellular gene silencing (Figure 2B; Chen et al., 2015). The long ssDNA obtained by RCA reaction served as the backbone chain, and two types of DNA nanoribbons were obtained with the DNA origami method in which designer short ssDNA was used as staple chains. Due to its relatively high aspect ratio and rigid structure, DNA nanoribbons could effectively enter lung cancer cells (H460) *via* endocytosis and escape from lysosomes, thus achieving efficient siRNA delivery and gene silencing. This study combined DNA origami technology with RCA reaction and provided an ingenious method for designing a siRNA delivery system. Liu et al. (2018) constructed a tailored DNA origami for synergistic RNA interference (RNAi)-/chemotherapy of multidrug-resistant tumors (Figure 2C). Triangular DNA origami with cleavable disulfide capture strands and targeting aptamers was constructed in one pot *via* DNA assembly, followed by the doxorubicin loading. Subsequently, the linear small hairpin RNA transcription template targeted to the multidrug resistance-related genes (P-glycoprotein and survivin) could be tethered on the capture strand *via* base pairing. The tailored DNA origami was internalized into breast cancer cells through mucin 1 (MUC1) targeting aptamer. With response to intracellular pH and glutathione (GSH), the DNA origami controllably released doxorubicin and gene drugs with a marked antitumor effect against multidrug-resistant tumors.

## DNA Hybrid Nanomaterials

To improve stability and introduce multiple functional units that are not provided by pure DNA nanomaterials, hybrid DNA nanomaterials have been widely investigated for nucleic acid drug delivery (Hendrikse et al., 2019). Hybrid DNA nanomaterials combined the intrinsic recognition capability of DNA with extra functions of other materials to improve the performance in nucleic acid drug delivery. For instance, introducing inorganic nanocomponents into the DNA nanosystem could achieve desired optical and electronic features; polymer or supermolecule with targeting and stimuli-responsive properties has been integrated with DNA to obtain spatiotemporally controlled drug assembly and release (Li F. et al., 2019). In this perspective, we summarized the developed hybrid DNA nanostructures including DNA-inorganic hybrids and DNA-organic hybrids for nucleic acid drug delivery. The challenges and opportunities of hybrid DNA nanomaterials in nucleic acid drug delivery were discussed.

### DNA-Inorganic Nanomaterials

**FIGURE 3:**
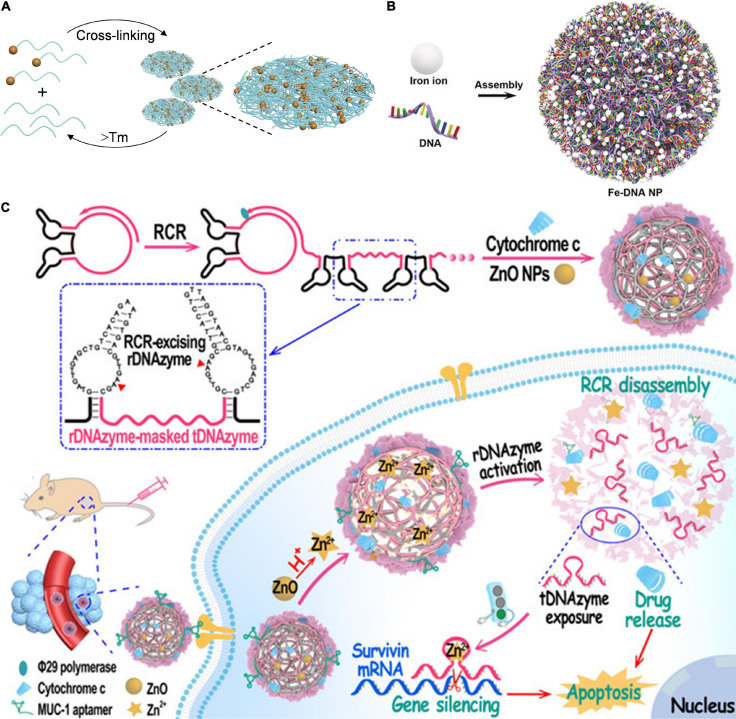
DNA-inorganic nanoassembly for nucleic acid-based drugs delivery. **(A)** Gold-DNA nanoassembly for efficient gene silencing with controllable transformation. Adapted with permission from ref. Huo and Gan (2019). Copyright 2019, Science Publishing Group. **(B)** Engineering Multifunctional DNA hybrid nanospheres through coordination-driven self-assembly for CpG delivery. Adapted with permission from ref. Li M. et al. (2019). Copyright 2019, Wiley-VCH. **(C)** DNA nanoflower for multifunctional DNAzyme delivery. Adapted with permission from ref. Wang et al. (2021). Copyright 2021, Wiley-VCH.

DNA could form hybrid nanomaterials with different moieties including inorganic NPs (Huo et al., 2019), mineral salt (Wang et al., 2021), and metal ions (Li M. et al., 2019). These DNA-inorganic hybrids are usually conjugated or electrostatically adsorbed with nucleic acid drugs (Singh et al., 2010). The inorganic nanoparticles could protect nucleic acids and improve their stability in plasma (Jiang and Thayumanavan, 2020). Take gold (Au) NPs as an example, ultrasmall (∼2 nm) AuNPs with surface functionalization could deliver oligonucleotides to directly attack the cellular nucleus with no need for any targeting moiety (Huo et al., 2014). Inspired by this, Huo et al. (2019) constructed gold-DNA nanoflowers *via* DNA-mediated self-assembly AuNPs for efficient gene silencing (Figure 3A). In their study, ultrasmall AuNPs were modified with cellular-myelocytomatosis viral oncogene (*c-myc*) silencing sequence (POY2T) which is a 23-nt (nucleotide) oligonucleotide *via* a ligand exchange method. Another designed ssDNA could complementarily hybridize with POY2T, thus blocking the *c-myc* oncogene silencing sequence *via* binding *c-myc* oncogene. Furthermore, POY2T modified AuNPs and the designed ssDNA would self-assemble into large-sized gold-DNA nanoflowers, which would dissociate and release ultrasmall AuNPs upon near IR (NIR) irradiation. This study fully utilized both the high nuclear internalization efficiency of ultrasmall AuNPs and enhanced tumor accumulation/retention of gold-DNA nanoflowers.

Metal-ligand coordination chemistry has emerged as another convenient strategy to synthesize nanomaterials with well-defined size and shape (Leininger et al., 2000). The key constituents of nucleic acids and nucleobases could coordinate with metal ions to form assemblies through coordination interactions (Amo-Ochoa and Zamora, 2014). For example, Li M. et al. (2019) reported a general approach for ferrous (Fe^2+^) ions directing the assembly of DNA hybrid nanospheres *via* the coordination interaction (Figure 3B). By adjusting the molar ratio of metal ions and DNA, Fe-DNA nanospheres with adjustable sizes and controllable functions were obtained driven by coordination interactions between Fe^2+^ ions and DNA due to the abundance of phosphate-binding sites and oxygen and nitrogen atoms on the nucleobases. Cytosine-phosphate-guanosine (CpG), which could produce cytokines to inhibit cancer cell proliferation, was used as a model functional nucleic acid in this system. However, the obtained NPs showed low stability in fetal bovine serum (FBS), limiting its biological applications. Then, the mineralized NPs with a zeolitic imidazolate framework-8 (ZIF-8) exoskeleton to enhance serum stability. Compared with naked CpG, Fe-CpG-ZIF-8 showed enhanced cellular uptake efficiency and higher bioactivities, demonstrating an efficient high DNA delivery.

Another typical kind of DNA-inorganic molecule hybrid is DNA nanoflower with a flower-like morphology produced with the RCA method. The DNA nanoflower could maintain the stability of DNA in the bloodstream and load additional metal ions to active the biological functions of DNA (Lv et al., 2020). As an efficient isothermal enzymatic reaction, RCA could produce long preprogrammed DNA chains stabilized with pyrophosphate. By virtue of these programmability and intrinsic biocompatibility of DNA motif, DNA nanoflowers showed great potential in nucleic acid drug delivery. For example, Liu group constructed a DNA aptamer and hypoxia-inducible factor 1α (HIF-1α) antisense DNA-integrated DNA nanoflower *via* RCA (Pan et al., 2020). The DNA nanoflower effectively inhibits HIF-1α protein expression, demonstrating efficient HIF-1α antisense DNA delivery *via* DNA nanoflower. Wang et al. (2021) reported a bioinspired self-catabolic DNA nanosponge for efficient gene silencing *via* the rolling circle replication (RCR) (Figure 3C). The therapeutic DNAzyme (tDNAzyme) and self-motivated DNA-excising DNAzyme (rDNAzyme) were designed as a template of RCR. tDNAzyme was masked by rDNAzyme during the RCR process. The obtained RCR scaffolds were modified with multivalent DNA aptamers (MUC1 aptamer), which could specifically target tumor cells. Besides, zinc oxide (ZnO) NPs (acting as DNAzyme cofactor precursors) and proapoptotic cytochrome c protein (acting as chemotherapy agents) were encapsulated in the DNAzyme nanosponge. This multifunctional nanosponge was internalized into targeted tumor cells by virtue of MUC1 aptamer strand, in lysosome the encapsulated ZnO NPs were translated into zinc ion (Zn^2+^), and the released Zn^2+^ could trigger the disassembly of rDNAzyme, leading to burst release of both rDNAzyme and cytochrome c for combined gene silencing and chemotherapy. This study fully combined DNA-inherent programmability and ZnO NP-promoting DNAzyme catalytic efficiency for enhanced DNAzyme-based therapy.

### DNA-Organic Nanomaterials

Biomolecules, such as peptides (Chin et al., 2018), proteins (Pelegri-O’Day and Maynard, 2016), and nucleic acids (Pan et al., 2017) could be integrated with synthetic polymers to form DNA-polymer hybrid nanomaterials with desirable functions (Luckerath et al., 2020). Compared with peptides and proteins, nucleic acids, such as precise biopolymers have unparalleled sequence programmability; therefore, the combination of nucleic acids with synthetic polymers could generate a variety of hybrid materials with extensive applications, especially in nucleic acid delivery (Luckerath et al., 2020).

**FIGURE 4:**
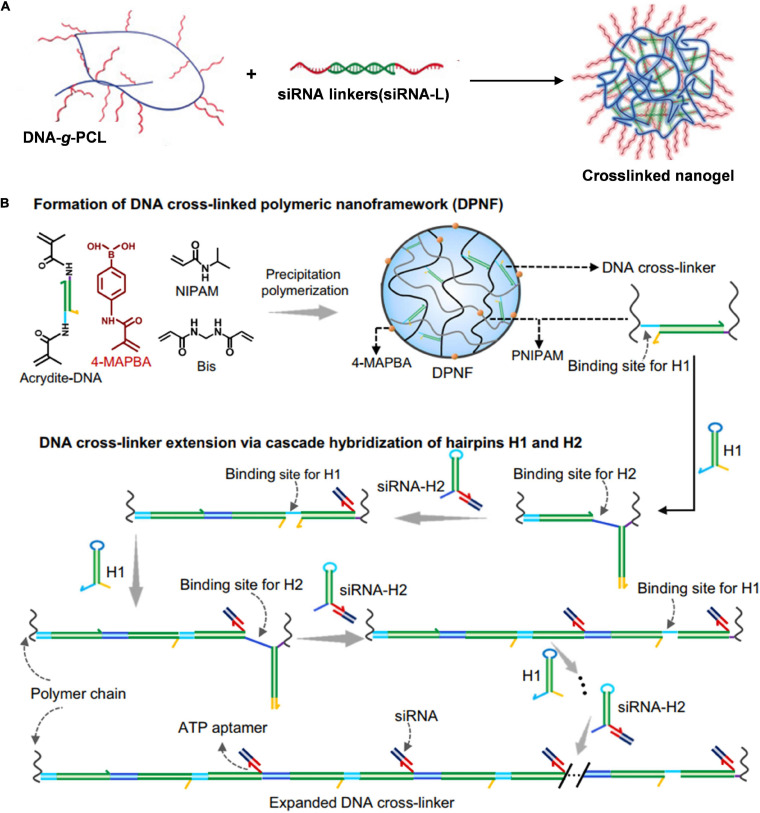
DNA-polymer nanoassembly for efficient siRNA delivery. **(A)** Crosslinked nucleic acid nanogel for effective siRNA delivery. Adapted with permission from Ding et al. (2018a). Copyright 2018, Wiley-VCH. **(B)** Cascade hybridization of hairpin DNA in polymeric nanoframework for precise siRNA delivery (Li et al., 2021). Copyright 2021, Nature Publishing Group.

In previous studies, siRNAs delivered by DNA tetrahedron were commonly exposed outside the nanostructures, which would inevitably make it degraded by ribonuclease (RNase). Conjugating nucleic acids with synthetic polymers could protect them from enzymatic degradation and prolong blood circulation. Ding et al. (2018) fabricated a cross-linked nucleic acid nanogel by using functional siRNAs as cross-linkers to direct self-assembly of DNA-grafted polycaprolactone (DNA-*g*-PCL) for effective siRNA delivery (Figure 4A). In their system, the siRNA was decorated with two single-stranded overhangs, which were complementary to the grated DNA in DNA-*g*-PCL, resulting in embedding siRNAs in the nanogel to avoid enzymatic degradation. The nanogel exhibited great knockdown efficiency of serine/threonine-protein kinase 1 (PLK1) in tumor tissues due to the enhanced cellular uptake. Moreover, the nanogel showed prolonged blood circulation with a half-life of ca. 1.14 h, which was much longer than those of naked siRNA (ca. 4 min) and **Lipofectamine** 2000 (Lipo2000) siRNA (ca. 10 min).

We achieved controlling DNA assemble under polymeric nanoconfinement and develop a dynamic DNA assembly strategy in the confined space of polymer nanometers for precise siRNA delivery (Figure 4B; Li et al., 2021). Initially, we prepared DNA cross-linked polymeric nanoframeworks (DPNFs) on which tumor-targeting phenylboronates were decorated *via* precipitation polymerization. Cross-linked DNA served as the initiator that could drive cascade hybridization chain reaction (HCR) where hairpin monomers used potential energy to overcome the steric hindrance of DPNFs. All of the self-assembly processes were isothermal enzyme-free amplification. The sticky end of one DNA hairpin was designed elaborately as a triphosadenine (ATP) aptamer for efficient siRNA delivery in the DPNFs *via* base complementary pairing. Furthermore, in response to intracellular abundant ATP, siRNA was released especially in the cellular cytoplasm. Moreover, the functional group phenylboronate could endow tumor-targeting nanocarriers of DPNFs with actively recognizing the overexpressed sialic acid residues on the tumor cell membrane, thus improving the *in vivo* efficiency of gene delivery.

In contrast to chemical covalent bonds, supramolecular host-guest interactions provide another method for molecular recognition through non-covalent interactions with reversibility and stimuli-responsive nature (Hu et al., 2018). β-cyclodextrin (β-CD), the glucose heptamer, was a food additive approved by the Food and Drug Administration (FDA) (Ashton et al., 1996; Davis and Brewster, 2004). As one of the best supramolecular “host” molecules, β-CD could combine with adamantane (Ad), which is a typically “guest” molecule to form inclusion complexes due to the efficient molecular recognition and relatively strong binding interaction between β-CD and Ad (Davis and Brewster, 2004). Reji Varghese group reported a series of nano-to-micro-sized DNAsomes through amphiphilicity-driven self-assembly (Thelu et al., 2017). These DNAsomes exhibited remarkable molecular recognition between β-CD-functionalized DNA (acting as the host molecule) and Ad-grafted hydrophobic segment (acting as the guest molecule). Subsequently, they further constructed nanogel with excellent cell permeability *via* self-assembly of β-CD-modified X-DNA or Y-DNA and Ad-grafted eight-arm star polyethylene glyco (PEG) polymer, showing its great potential in nucleic acid drugs delivery. Ding group have made great progress in the field of multifunctional nucleic acid nanostructures to deliver gene therapy drugs.

**FIGURE 5:**
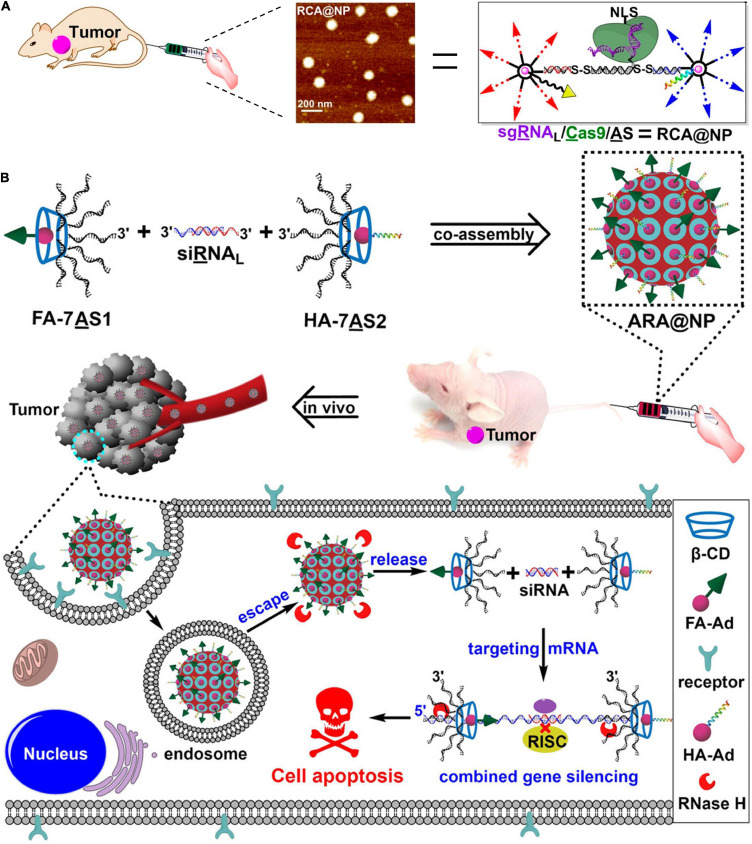
DNA-supermolecule nanoassembly for nucleic acid delivery. **(A)** Multifunctional nucleic acid nanostructures for sgRNA/Cas9/antisense delivery. Adapted with permission from ref. Liu et al. (2019). Copyright 2019, American Chemical Society. **(B)** Crosslinked nucleic acid nanogel for effective siRNA delivery. **(B)** Co-assembly of branched antisense and siRNA for combined gene silencing and tumor therapy. Adapted with permission from ref. Liu et al. (2021). Copyright 2020, Wiley-VCH.

In 2019, Ding et al. (2019) further utilized tile-mediated assembly of β-CD-Ad and branched DNA to construct DNA-supermolecule hybrid nanostructure, achieving sgRNA/Clustered regularly interspaced short palindromic repeats associated 9 (Cas9)/antisense DNA delivery (Figure 5A). They used the circular supramolecular β-CD as the core to covalently cross link nucleic acids by virtue of copper-free click chemistry to prepare branched DNA with seven-arm structures. Meanwhile, the 3′-end of the sgRNA (targeting the tumor-related gene PLK1) was extended to construct an RNA strand (sgRNA_*L*_), which is complementary to ASO. Antisense (AS, targeting the tumor-related gene PLK1) modified by disulfide bonds at both ends was introduced as a linker for coassembly of the gene editing component (sgRNA/Cas9) and the gene silencing component (AS). An Ad-conjugated aptamer and Ad-modified hyaluronic acid (HA) endowed the nanoplatform with tumor-targeting and endosomal escape capabilities through host-guest interaction, respectively. Subsequently, sgRNA/Cas9/AS dissociated into sgRNA/Cas9 complex and antisense in response to intracellular GSH and RNase H, exhibiting excellent PLK1 gene silencing and potent antitumor effect. Also, based on this strategy, a coassembled nanoplatform of branched antisense DNA and siRNA was constructed for combined gene silencing *in vitro* and *in vivo* (Figure 5B; Liu et al., 2021). Covalently binding branched antisense can effectively capture the 3′-end siRNA to form NPs with controllable size. Second, biocompatible nucleic acid nanostructures can be functionalized with targeting groups and endoplasmic escape components through host-guest interactions. Finally, multifunctional nucleic acid nanocarriers can be digested by endogenous RNase H to effectively release nucleic acid products (branched antisense and siRNA). This is the biocompatible multifunctional nucleic acid nanoplatform. This novel strategy used skillfully nucleic acid self-assembly and host-guest interaction to combine DNA with peptide, realizing the multi-functionalization of the nucleic acid delivery vector and providing a new frontier of nucleic acid delivery.

**FIGURE 6:**
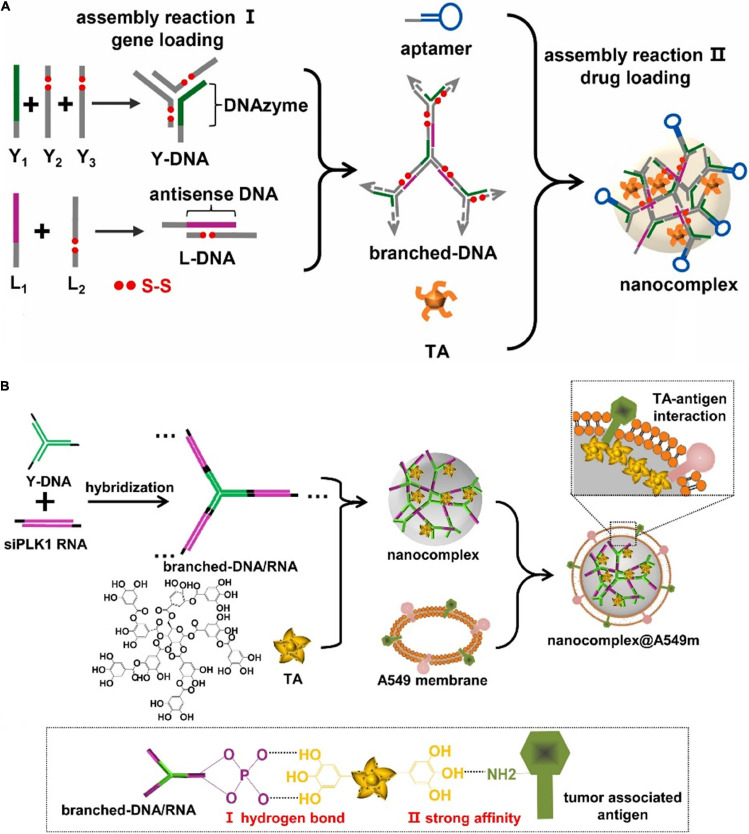
Nanoassembly *via* TA mediated nucleic acid assembly. **(A)** DNA-polyphenol nanocomplex for efficient DNAzyme and antisense DNA delivery. Adapted with permission from ref. Han et al. (2021a). Copyright 2021, Elsevier Publishing Group. **(B)** Nucleic acid nanocomplex through TA mediating self-assembly for smart drug delivery and gene therapy. Adapted with permission from ref. Han et al. (2021b). Copyright 2021, Elsevier Publishing Group.

Natural polyphenol originated from bioactive components of plant-based food. The typical biological role of polyphenol is reflected in the therapeutical functions for cancer therapy, such as anti-inflammation, anti-oxidation, and anti-apoptosis. In addition, the structural features of polyphenol also endowed strong interactions with nucleic acids to fabricate DNA-organic formulations, which demonstrated sufficient loading efficiency of gene drugs. In this regard, we employed tannic acid (TA) to mediate the assembly process of the dynamic nanocomplex system (Figure 6A; Han et al., 2021a). In the composite system, Y-shaped DNA and linear DNA assembled into branched skeleton through complementary base pairing and formed nanocomposites with TA driven by hydrogen bonding. The acidic environments in the lysosome would induce the degradation of TA and resulted in the disassembly of the nanocomplex to release branched DNA to the cytoplasm. In response to intracellular GSH and DNase I, branched DNA was cleaved to antisense DNA and DNAzyme segments, which afterward suppressed cell proliferation and cell migration, respectively. Notably, TAs also facilitated cell apoptosis to enhance the therapeutic effects. DNA-polyphenol coordinated nanocomplex ensured extremely high loading efficiency and achieved the responsive and sequential release of gene/drugs. The incorporation of TAs into the structural composition offered adequate controllability and flexibility in the design of drug delivery carriers, which showed excellent efficacy at *in vivo* levels.

Despite the diverse drug nanocarriers, the limited targeting specificity and blood stability generally resulted in poor enrichments to tumor sites. Inspired by the camouflage mechanism of cancer cells against the immune system, the cell membrane coating strategy provided feasible means to manipulate the fates of original nanocarriers. Due to the structural affinity between TAs and proteins, we further coated DNA-polyphenol nanocomplex with the cell membrane (Figure 6B; Han et al., 2021b). The homotypic targeting capability derived from the membrane improved the blood circulation stability of nanocomplex and reduced the clearance by the reticular endothelial system. In the cancer cells, the acid-responsive degradation served as the trigger for the decomposition of the nanocomplex, which further led to the controllable release of the functional nucleic acid moiety. In this dynamic complex system, TAs connected the outer shell of the cell membrane and inner nucleic acids components, which realized efficient drug delivery while demonstrated specific and on-demand release behaviors. TAs as the natural polyphenol are arguably promising candidates for engineering intelligent drug delivery platform in the field of precision medicine.

## Conclusion and Outlook

In this study, we have summarized the recent progress in DNA functional nanostructures for the delivery of nucleic acid-based drugs. Due to the sequence programmability of DNA, DNA-based nanostructures are highly designable at the molecular level, which facilitates to realize controllable assembly and disassembly of nucleic acid-based drugs in specific application scenarios. Furthermore, DNA molecules are easily combined with other assembly systems to achieve functional scalability and thus to improve delivery performances. Despite the rapid development in DNA-based gene delivery nanomaterials, the clinical transformation of those approaches still confronted with several challenges, including standardized manufacture, stability, biosafety concerns, and socioeconomic issues.

The standardized manufacture of DNA-based gene delivery nanomaterials needs further exploration. Although DNA has controlled structural conformation and sequence programmability, the synthetic efficiency of DNA assembly is still relatively low due to steric hindrance and limited atom utilization, which impedes standardized mass production. Optimizing and controlling the assembly condition might be an avenue to improve the efficiency of constituent atom utilization.

To enhance the delivery and therapeutic efficacy, DNA-based nanomaterials need prolonged blood circulation time to achieve efficient accumulation at lesion sites. However, the prolonged circulation time may cause degradation concerns *in vivo*. Therefore, the stability of DNA nanomaterials is vital for the effective delivery of nucleic acid-based drugs. Chemical modifications of DNA and integration with other materials have been proposed to be effective to enhance resistance to enzymatic degradation and improve the stability of DNA-based nanomaterials.

DNA is an endogenous substance with excellent biosafety and biocontrollable degradability. Studies have shown good biosafety of DNA-based nanomaterials, whereas limited investigation has involved long-term biosafety issues. Moreover, immunostimulatory sequence-integrated DNA nanomaterials may induce immune responses. Rational DNA sequence design of DNA-based nanomaterials is needed to guarantee the long-term biological security of DNA nanomaterials. However, there might be immune responses toward DNA, which is hard to completely avoid on occasions.

DNA used for the fabrication of DNA nanomaterials is mainly chemically synthesized currently. However, the length of DNA is limited to below 200 nucleotides. Therefore, the high cost of DNA-based nanomaterials remains a major challenge, which could cause socioeconomic issues to satisfy large-scale production for nucleic acid delivery. Exploring more effective methods to synthesize DNA and developing cost-effective storage approaches are highly necessary. The techniques to obtain biomass DNA may address this cost concern. For example, the plasmid containing target DNA fragments is transfected into bacteria, such as *Escherichia coli (E. coli)* to amplify DNA along with the growth of the bacteria. Another available technique is using PCR to amplify specific DNA fragments, which would reduce the cost of DNA production.

In summary, it is firmly believed that nucleic acid drug therapeutics are doomed to generate a revolutionary impact on disease treatment in the near future.

## Author Contributions

ZL, YZ, and FL: writing – original draft preparation. ZL and FL: writing – review and editing. FL: supervision. All authors contributed to the article and approved the submitted version.

## Conflict of Interest

The authors declare that the research was conducted in the absence of any commercial or financial relationships that could be construed as a potential conflict of interest.

## Publisher’s Note

All claims expressed in this article are solely those of the authors and do not necessarily represent those of their affiliated organizations, or those of the publisher, the editors and the reviewers. Any product that may be evaluated in this article, or claim that may be made by its manufacturer, is not guaranteed or endorsed by the publisher.
